# Development and Validation of a Method for the Analysis of Zinc Oxide in Cosmetic Matrices by Flame Atomic Absorption Spectroscopy

**DOI:** 10.1155/2021/8840723

**Published:** 2021-05-28

**Authors:** Luis F. Benavides, Juan D. Marín, Cristian Rosales, Johnbrynner García

**Affiliations:** Belcorp Research and Development, Tocancipá 251017, Colombia

## Abstract

A fast and simple method for the extraction and Flame Atomic Absorption Spectroscopy (FAAS) quantification of ZnO in different cosmetic matrices, including lipsticks, water-in-oil foundations, and oil-in-water creams, was developed and validated, according to the International Council for Harmonization of Technical Requirements for Pharmaceuticals for Human Use (ICH) and the United States Pharmacopeial Convention guidelines. The sample preparation consisted of an ultrasound-assisted ethanolic extraction of ZnO followed by digestion with 1 M nitric acid (HNO_3_). Samples were analyzed by Flame Atomic Absorption Spectroscopy (FAAS). Specificity, linearity, the limit of detection (LOD), the limit of quantification (LOQ), sensitivity, precision, and accuracy parameters were studied. The robustness of the method was evaluated with a five-variable Youden–Steiner model. The method was specific for ZnO, and the extraction procedure did not affect the stability of the signal compared to the background. The method was linear in the range 0.2–1.0 mg/L with LOD/LOQ values equal to 0.0156 (mg·L^−1^)/0.0473 (mg·L^−1^), 0.0098 (mg·L^−1^)/0.0297 (mg·L^−1^), 0.0113 (mg·L^−1^)/0.0341 (mg·L^−1^), and 0.0131 (mg·L^−1^)/0.0397 (mg·L^−1^), respectively, for raw material, lipstick, liquid foundation, and emulsion matrices. Regarding precision, the %RSD values were below 3.0% for repeatability and intermediate precision. Global reproducibility RSD was below 8.0% for all matrices. The percentage of recovery was not statistically different from 100% in all cases. The final concentration was found to be a critical variable for all matrices except for the raw material. The variables associated with the extraction step (ethanol volume, bath temperature, and extraction time) were critical in the extraction of liquid foundations and cream emulsions. The method reduces the number and concentration of mineral acids spent on the digestion of ZnO, and its application is extendable to raw materials. This development is an adequate tool for routine analysis and cosmetic quality control of chemically different products that contain ZnO as ultraviolet radiation (UV) filter, to guarantee regulatory compliance and ensure the safety and efficacy of products delivered to consumers.

## 1. Introduction

Cosmetics have played an important role in our society since ancient times until today, driven in part by a constantly evolving innovation culture [1, 2]. With ongoing and almost uninterrupted growth, the cosmetic market size was estimated at € 220 billion in 2019. Remarkably, skincare accounts for 40% of the total market size, well ahead of haircare (21%) and makeup (18%) segments [3]. Nowadays, innovation in those segments is concerned with the development of multifunctional products with appealing properties [4]. Sun protection stands out as a characteristic no longer limited to sunscreens, and the number of consumers that use sun protection products before sun exposure has increased in recent years [5]. It has become a desirable and almost necessary feature in products for skincare and makeup routines, increasing the use of ingredients that act as UV filters in their formulas [6].

UV filters are intended to prevent the long known adverse effects of UV radiation on the skin, among which melanoma is one of the most important [7]. UV radiation comprises a range of frequencies that can be subdivided into UVA (lowest energy, 320–420 nm), UVB (middle span, 280–320), and UVC (highest energy, 100–280 nm) [8]. Sun protection products are mainly designed to block UVA and UVB types, as UVC is mostly absorbed by oxygen to produce ozone in the upper atmosphere and is not able to come to Earth's surface. Because of its longer wavelength, UVA can deeply penetrate the skin reaching the dermis and causing DNA damage by the generation of reactive oxygen species (ROS), which induce oxidative base modifications and long-term effects as premature aging. On the other hand, UVB rarely penetrates beyond the epidermis and is associated with the most frequent short-term damage to the skin, known as sunburn, and some types of DNA damage as pyrimidine dimer formation and replication errors. Both UVA and UVB cause erythema and increase the risk of melanoma [9].

To cover the broad spectrum of UV radiation, multiple UV filters of different chemical nature are included in sun protecting formulas. These photoprotective agents can be classified, based on their composition, as organic or inorganic. In general, organic filters block UV radiation by absorbing photons whereas inorganic agents absorb, reflect, and scatter them [8]. A wide variety of organic molecules confer protection from UVA (e.g., benzophenones and avobenzone), UVB (e.g., PABA derivatives, cinnamates, salicylates, octocrylene), or both (wide spectrum filters as Ecamsule and Silatriazole). Inorganic UV filters include kaolin, talc, calcium carbonate (CaCO_3_), magnesium oxide (MgO), titanium dioxide (TiO_2_), and zinc oxide (ZnO) [10]. Recently, the role of inorganic filters in the formulation of sun protection products has gained importance as organic filters tend to be more harmful to the environment [11].

ZnO and TiO_2_ are approved by the U.S. Food and Drug Administration (FDA) as photoprotective agents [12]. ZnO is more effective in protecting from UVA and TiO_2_ from UVB; hence, a combination of both gives cosmetic formulas wide spectrum protection [13]. However, one major drawback of using these minerals in some cosmetic products is the unwanted white, milky appearance they leave on the skin after application. To solve this issue, nanosized ZnO and TiO_2_ materials have been developed that suppress this effect while maintaining their UV-blocking properties [14], but the bioreactivity of nanosized ingredients in cosmetic products is not free from scrutiny, and some safety and environmental concerns have been raised [11]. Despite this, ZnO and TiO_2_ nanoparticles are regarded as safe ingredients in cosmetic formulas. Whether used as nanoparticles or in bigger particle sizes, they are allowed up to a limit of 25% w/w [12]. Consequently, its quantification is important not only for ensuring the safety and efficacy of cosmetic products but also for guaranteeing regulation compliance [15].

Metal analysis, specifically, Zn quantification via atomic absorption spectroscopy (AAS), is widely used because of its practicality and low cost. It is well documented in matrices such as biological, food, and water samples [16–22]. Different approaches for the extraction or preconcentration of the metal are performed among which are included liquid-liquid extractions with ionic liquids [20] and ternary solvent systems [23], solid-phase extractions with modified activated carbon [19] and modified Amberlite resins [21], and classic approaches involving acid digestion [18, 22]. Preconcentration steps are normally required when the amount of zinc in a working quantity of the sample is lower than the limit of detection of the method. These preconcentration steps generally belong to methods where the primary interest is quantifying metals to ensure that they are below toxic levels or in trace amounts. On the other hand, methods involving acid digestions assisted with energy sources such as conventional heating or microwave and ultrasound-assisted digestions are suitable for the analysis of metals in higher concentrations and from the matrix of more complexity such as cosmetic matrices.

Only a small fraction of the literature dedicated to UV filter analysis in cosmetic matrices is devoted to the quantification of inorganic filters, especially of ZnO [15]. ZnO analysis in cosmetic formulas via different analytical techniques as complexometric titration [24], ion-exchange chromatography (IC) [25], square wave voltammetry [26], atomic absorption spectroscopy [13, 27, 28], and inductively coupled plasma atomic absorption spectroscopy (ICP-AES) [2, 29] has been reported. Considering methods mainly focused on ZnO quantification, the general AAS/FAAS approach to digest ZnO uses mild to strong mineral acids as HF, HCl, HNO_3_, or their mixtures [18, 29, 30]. A source of external energy as conventional heating [15, 29] or microwave-assisted heating [15, 26] is also employed to ensure that the digestion reaction goes to completion.

Different cosmetic products can have different base formulas where the ratio of lipophobic to lipophilic ingredients, the presence of thickening agents, or the presence of structuring solids varies considerably [31]. For that reason, they may benefit either from simplified methods with milder conditions or from more aggressive methods suitable for their composition. For instance, extraction from a water-based system can be performed under relatively mild conditions with aqueous solutions of diluted acids or even in their absence [13, 29]. Conversely, thick, oily products or possibly waxy solids might need an extraction step with organic solvents like diethyl ether [24, 32] or acetonitrile [25] to release the ZnO from the matrix. Development and validation of methods specialized for every kind of cosmetic product represent an important investment of both resources and time, making the development of a method with a wide scope ideal. To the best of our knowledge, no validated method for ZnO extraction and quantification tested in different cosmetic matrices has been reported.

In this work, we aim to develop and validate a simplified method for the routine extraction and quantification of ZnO in different cosmetic matrices by FAAS, assessing its specificity, linearity, precision, accuracy, and robustness according to ICH and United States Pharmacopeia guidelines [33, 34]. To meet this purpose, a cream emulsion, a liquid foundation, and a lipstick were selected because of their chemically different base formulas. The method is intended as an easy-to-perform and cost-effective solution for the quantification of ZnO in chemically different cosmetic products that include this ingredient as a UV filter and in raw materials to evaluate its purity.

## 2. Materials and Methods

### 2.1. Reagents and Materials

Zinc 1000 mg/L stock solution (Centipur ®), analytical grade 65% HNO_3_ (EMSURE ®), and analytical grade absolute ethanol (EMSURE ®) were purchased from Merck (Darmstadt, Germany). ZnO raw material (UV-Cut-ZnO-61-DM™) was obtained from Grant Industries (New Jersey, USA). Lipsticks, liquid foundations, and cream emulsions were obtained from Belcorp (Lima, Perú). Deionized water (resistivity ≥ 18 MΩ *∗* cm) was prepared with a Barnstead™ Easy Pure™ II water purification system.

### 2.2. FAAS Conditions

A Thermo Scientific™ iCE™ 3000 atomic absorption spectrometer with an air-acetylene flame where acetylene flux was adjusted as 1.2 (L·min^−1^) was used to perform the measurements of Zn absorbance. A Hollow Cathode Lamp at a wavelength of 213.9 nm with a bandpass of 0.2 nm was used. Background correction was achieved with a deuterium lamp.

### 2.3. Sample Collection

Product samples of lipstick, foundation, and emulsion were randomly chosen for ZnO analysis from industrial-scale batches produced in Belcorp, Tocancipá, Colombia. The raw material (UV-Cut-ZnO-61-DM™) was analyzed as delivered by the supplier. Placebo stocks were prepared using the same list of ingredients from lipstick, foundation, and emulsion product formulas, but removing the ZnO raw material from the preparation.

### 2.4. Sample Preparation

Calibration standards were prepared by diluting an adequate amount of Zinc stock solution with 1 M HNO_3_. To prepare placebo stock solutions, a weighted amount (40 mg lipstick or foundation, 80 mg emulsion) of placebo was placed in a 100 mL volumetric flask. A 70 mL volume of absolute ethanol was added, and the flask was mechanically stirred for 1 min using an MS1 vortex mixer (IKA, Staufen, Germany). After being vortexed, the content was extracted by sonication at 50°C for 20 min. Later, a 20 ml volume of 1 M HNO_3_ acid was added to the flask, and a second sonication step was performed under the same conditions. Upon cooling to room temperature, the mixture was further diluted to volume with 1 M HNO_3_ acid. The resultant solution was filtered through a 0.45 *μ*m nylon syringe filter. Placebo standards were prepared by transferring 1 mL of this solution to a 50 mL volumetric flask and adding the appropriate amount of Zinc standard solution. The contents were diluted to volume with 1 M HNO_3_ acid. Cosmetic and raw material samples were prepared by placing an accurately weighed amount, between 10 mg and 500 mg, in a 100 mL volumetric flask. After extracting its contents using the same protocol as in placebo extractions, the resultant mixture was filtered with a 0.45 *μ*m nylon syringe filter. 1 ml of the filtered solution was transferred to a 100 mL flask and diluted to volume with 1 M HNO_3_ acid for FAAS analysis.

### 2.5. Method Validation

The validation parameters specificity, linearity, LOD, LOQ, repeatability, intermediate precision, global precision, accuracy, and robustness were considered based on current directives as outlined by ICH and United States Pharmacopeia guidelines [33, 34].

#### 2.5.1. Specificity

The extraction method was performed on placebos (samples without ZnO) to yield solutions that bore similar chemical characteristics as the cosmetic samples, named placebo stocks, to which no Zn standard was added. Duplicate samples of each blank were prepared. The method specificity was considered as the ability to differentiate the Zn signal from the signal of the background and the signals of the matrices.

#### 2.5.2. Linearity and Sensitivity

For each cosmetic matrix and the raw material, three calibration plots using 0.2, 0.4, 0.6, 0.8, and 1.0 ppm standards were prepared to evaluate the linearity of the method. The statistical significance of the linear regression was assessed using analysis of variance (ANOVA), and the correlation was evaluated using a Student's *t*-test. The sensitivity of the method was estimated by finding the limit of detection (LOD) and the limit of quantification (LOQ) from the regression data. LOD was calculated with formula 3 *∗* (*σ*/S) and LOQ with formula 10 *∗* (*σ*/*S*), *σ* being the standard error of the intercept and S the slope of the regression equation. Six replicates of stock dilutions/placebo stock dilution were measured to validate LOQ by estimating its accuracy and precision. An upper limit of 5.0% for RSD was set for those measurements. Product samples of lipstick, foundation, and emulsion were analyzed to determine their ZnO content using the method developed in this study.

#### 2.5.3. Precision

The precision of the method was evaluated by estimating its repeatability, intermediate precision, and reproducibility. For repeatability, six replicates at a low, medium, and high level of concentration, which were diluted from stocks to an approximate value of 3.2, 4.0, and 4.8% w/w for lipstick and 6.4, 8.0, and 9.6% w/w for foundation and emulsion (duplicate for each level), were measured. For the raw material, only six replicates of a central level equal to its nominal concentration (0.4% w/w as declared in the certificate of analysis) were analyzed. The upper limit for % RSD was set at 5.0% for these measurements. For intermediate precision, sample duplicates at the medium level were measured on two different days by two different analysts. %RSD upper limits for intraday and interday measurements were set at 5.0%, and for the global % RSD, the limit was set at 8.0%, as suggested by the United States Pharmacopeia validation criteria [34]. Reproducibility was evaluated in two different laboratories by measuring the raw material and three product samples whose nominal concentrations are 4% w/w for lipsticks and emulsions and 9% w/w for foundations.

#### 2.5.4. Accuracy

Accuracy was evaluated using solutions of placebo to which an adequate amount of zinc standard was added to obtain concentrations in the low, medium, and high levels described in the previous item. No addition of zinc standard was performed on raw material samples, as the concentration reported in its certificate of analysis was assumed as the nominal value. The recovery rate was calculated from the measurements of duplicate samples for each matrix.

#### 2.5.5. Robustness

A Youden–Steiner model [35] was applied to five critical variables of the method. Method standard conditions and alternative conditions were studied in eight experiments for each cosmetic matrix and the raw material. The experimental setup is outlined in Table 1. Duplicate samples were measured at each condition. Critical variables were those that exhibited an absolute difference greater than *σ* *∗* $\sqrt{2}$, where *σ* is the standard deviation derived from the repeatability assays.

## 3. Results and Discussion

### 3.1. Method Development

The cosmetic matrices considered in this work include lipsticks, which can be thought of as waxy matrices [36], to foundations, including but not limited to water-in-oil emulsions which contain dimethicone and dimethicone-derived emulsifiers [37, 38], and cream emulsions that include both water-in-oil and oil-in-water emulsions [4]. The diverse chemical properties of these matrices make the use of an extraction solvent necessary, as direct acid digestion might require long times for products with high lipophilic material content. Ethanol was selected and used in a first step to dissolve the cosmetic matrix under mild conditions in an ultrasound bath. This step is intended to release the ZnO from the matrix and, because of that, its duration, the volume of ethanol, and the temperature of the bath were considered for robustness assays, as discussed hereinafter. A second sonication step in the presence of relatively low-concentration HNO_3_ was devised to finally digest the ZnO. Because of the low concentration of the acid, compared to methods where it is used at 65% w/w, the step duration is important because it determines whether the digestion goes to completion or not; thus, it was also considered for robustness assays. The combination of these two steps significantly reduces the amount of acid spent per sample, without sacrificing the ability to extract ZnO from chemically different cosmetic products. The applicability of the method was extended to the quantification of ZnO in raw materials because it was reasoned that they can be considered the limiting case where matrix complexity is almost null.

### 3.2. Method Validation

#### 3.2.1. Specificity

**Figure 1:**
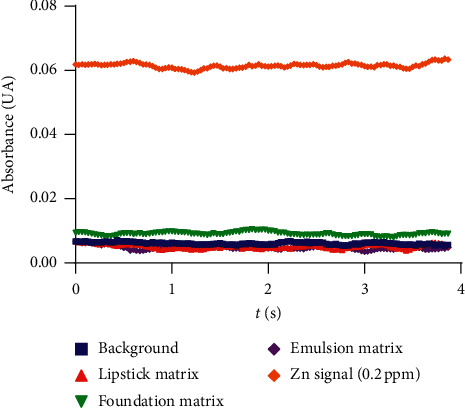
Absorbance as a function of time for background, placebo blanks, and Zn (0.2 ppm) signals.

Figure 1 presents a plot of the Zn signal at the lowest concentration used for the calibration plots (0.2 ppm) along with the background signal and the signals associated with each cosmetic matrix and raw material. The difference in the signal of Zn compared to the other signals indicates a successful remotion of potential interferences and shows that the method is able to differentiate Zn from excipients when the extraction is performed from the cosmetic matrices and the raw material considered in this study.

#### 3.2.2. Linearity and Sensitivity

For the quantification of ZnO in cosmetic raw materials, lipsticks, foundations, and emulsions, three calibration plots were obtained for each type of matrix. The data was adjusted using Least Squares Regression, and the suitability of the model was evaluated based on the regression coefficient as shown in Table 2. The values agree with the acceptance criterium defined for this parameter (*r*^2^ ≥ 0.995) and, consequently, it is possible to affirm that the model is well adjusted by a linear equation in the range of concentrations studied. Besides, these regressions were statistically significant, and the correlation found was significantly linear as demonstrated by ANOVA and Student's *t*-test results, both at a 0.05 significance level.

Calculated LOD and LOQ values are reported in Table 2. These results show that the sensitivity of this method is comparable to other atomic spectroscopy methods [28, 29]. LOQ precision was within the limits defined in this work, following United States Pharmacopeia guidelines (%RSD ≤ 5.0%) [34]. Regarding accuracy, the percentages of recovery at LOQ were not statistically different from 100%.

The ZnO average content of product samples and SD of three replicates are presented in Table 3. These cosmetic samples were chosen because even though they represent chemically different products, they possess a fixed amount of ZnO across their formulas (4% w/w for lipsticks and emulsions and 9% w/w for foundations). The found concentrations represent the true ZnO content which complies with current regulation guidelines [12, 15].

#### 3.2.3. Precision

Results for repeatability, intermediate precision, and reproducibility are shown in Table 4. For all cosmetic matrices in all three concentration levels and the raw material at its nominal concentration, a %RSD below 3.0% was obtained. Thus, good repeatability is observed under method conditions. Intermediate precision exhibits a similar trend, with %RSD values being no greater than 3.0% for all the cosmetic matrices. In general, the values are well below the %RSD limit defined by the authors and are comparable to results obtained for other techniques [25, 26] and types of matrices [39]. Global reproducibility results agree with the limit set for this method (%RSD ≤ 8.0%) and in most categories are below 5.0%.

#### 3.2.4. Accuracy

The accuracy of the method was evaluated by comparing the amount of ZnO recovered from each cosmetic matrix with its nominal concentration. Recovery rates are presented in Table 5. Statistical significance for the results was assessed with a Student's *t*-test at a 0.05 significance level, which showed that the recovery rates were not significantly different from 100%. %RSD values for individual concentration levels were below 3.0%, thus illustrating the low variability that can be achieved through the extraction method described in this work.

#### 3.2.5. Robustness

Ethanol volume, time of first sonication step, time of second sonication step, ultrasound bath temperature, and final concentration were studied under a Youden–Steiner model, defining method predetermined variable values as standard conditions and deviations from those standard values as alternative conditions.  Table 6 shows the results for each variable under a given cosmetic matrix and their corresponding critical value. As can be seen from these results, the raw material matrix analysis is unaffected by small variations in any of the variables studied. This finding could be explained by the simplified nature of the raw material matrix, but also because of its high ZnO content (around 50% w/w), compared to the content of the other matrices, which positively affects sample preparation variability. Conversely, in lipstick, foundation, and emulsion matrices' analysis, final concentration was observed to be a critical variable, which can be linked to the low content of ZnO in their formulas (below 10% w/w) and, therefore, to increased variability in sample preparation. Other critical variables were ethanol volume and first sonication step time for the emulsion matrix and ultrasound bath temperature for the foundation matrix. These results show that the first extraction step is critical for the outcome in the analysis of emulsions and foundations. Consequently, special care to ensure conditions like those predetermined by the method should be taken.

## 4. Conclusions

A method for the extraction and FAAS quantification of ZnO in cosmetic products was developed and validated according to ICH and United States Pharmacopeia guidelines. The method is specific, accurate, precise, and robust. It allows the extraction of ZnO from several cosmetic matrices that differ in their chemical composition, with no need for high concentration mixtures of mineral acids. Because of its versatility, this method can be extended to the quantification of ZnO in raw materials. This method provides a tool for the routine analysis and cosmetic quality control of ZnO in different products that must comply with FDA, European Commission, and other applicable regulations to guarantee the safety and efficacy of the products delivered to the consumer.

## Figures and Tables

**Table 1:** Difference values for the variables studied under the Youden–Steiner model.

Parameter	Difference			
	Raw material	Lipstick	Foundation	Emulsion
Ethanol (mL)	0.56	0.07	0.07	**0.21**
Ultrasound bath temperature (°C)	0.37	0.07	**0.15**	0.02
First sonication step (min)	0.09	0.01	0.04	**0.17**
Second sonication step (min)	0.37	0.00	0.04	0.03
Final concentration (ppm)	0.29	**0.13**	**0.24**	**0.24**
Critical value	0.974	0.093	0.128	0.159

Bold values correspond to critical variables.

**Table 2:** Linearity and sensitivity parameters for all cosmetic matrices.

		Raw material	Lipstick	Emulsion	Foundation
Linear range	Slope	0.3502	0.2694	0.2539	0.2635
	*r* ^2^	0.9979	0.9992	0.9989	0.9985
	*t* value^a^	128.2	204.2	177.7	152.5
	*F* value^b^/10^4^	1.64	4.17	3.16	2.33

Sensitivity	LOD (mg·L^−1^)	0.0156	0.0098	0.0113	0.0131
	LOQ (mg·L^−1^)	0.0473	0.0297	0.0341	0.0397
	(i) %RSD	2.97	2.46	2.01	1.95
	(ii) %recovery	99.4	99.5	99.9	101.1
	(iii) *t* value^a^	0.509	0.492	0.122	1.41

^a^ *t* critical value (0.05 significance level): 2.032 (linearity) and 2.571 (LOQ). ^b^*F* critical value (0.05 significance level): 4.1366.

**Table 3:** ZnO content in samples of lipstick, foundation, and emulsion products.

Sample	Average found concentration (% w/w ± SD)
Lipstick 1	3.5341 ± 0.0137
Lipstick 2	3.5994 ± 0.0058
Lipstick 3	3.541 ± 0.0001

Foundation 1	8.7116 ± 0.0311
Foundation 2	8.8452 ± 0.0062
Foundation 3	8.8172 ± 0.0241

Emulsion 1	3.8630 ± 0.0030
Emulsion 2	3.7872 ± 0.0001
Emulsion 3	3.7656 ± 0.0192

**Table 4:** Average found concentration, standard deviation, and total variation (%RSD) for the precision levels considered in this study.

		Raw material	Lipstick	Emulsion	Foundation
Repeatability (mean ± SD% w/w; %RSD)	Level 1		3.155 ± 0.053; 1.69	6.629 ± 0.130; 1.95	5.593 ± 0.120; 2.15
	Level 2	59.13 ± 0.69; 1.16^a^	3.954 ± 0.066; 1.66	8.796 ± 0.113; 1.28	7.793 ± 0.091; 1.16
	Level 3		4.627 ± 0.076; 1.64	9.784 ± 0.133; 1.36	9.613 ± 0.136; 1.42

Global intermediate precision (mean ± SD% w/w; %RSD)	Day 1	56.16 ± 0.73; 1.30	3.628 ± 0.067; 1.85	8.310 ± 0.120; 1.45	8.469 ± 0.290; 3.43
	Day 2	58.09 ± 0.77; 1.32	3.810 ± 0.036; 0.95	8.443 ± 0.071; 0.84	8.577 ± 0.146; 1.70
	Global	57.13 ± 1.23; 2.16	3.719 ± 0.108; 2.91	8.377 ± 0.117; 1.40	8.523 ± 0.226; 2.65

Global reproducibility (mean ± SD% w/w; %RSD)	Laboratory 1	59.55 ± 0.33; 0.55	3.722 ± 0.055; 1.47	3.517 ± 0.022; 0.64	8.697 ± 0.051; 0.58
	Laboratory 2	56.80 ± 1.36; 2.39	4.025 ± 0.166; 4.12	3.673 ± 0.089; 2.42	8.983 ± 0.095; 1.05
	Global	58.17 ± 1.75; 3.01	3.873 ± 0.200; 5.15	3.595 ± 0.103; 2.88	8.840 ± 0.171; 1.93

^a^Raw material was evaluated at its nominal concentration, as declared by the certificate of analysis.

**Table 5:** Recovery rates for all the cosmetic matrices studied in this work.

	Raw material^c^	Lipstick			Emulsion			Foundation		
Added concentration (% w/w)	60.4	3.170	3.960	4.750	6.560	8.520	9.600	5.670	7.980	9.670
Average found concentration^a^ (% w/w, %RSD)	59.554, 0.51	3.186, 1.69	3.989, 0.49	4.639, 1.44	6.543, 1.19	8.728, 1.08	9.702, 1.01	5.663, 1.43	7.860, 0.95	9.640, 1.90
Recovery rate (mean ± SD)	98.66 ± 0.54	100.60 ± 1.89	100.86 ± 0.31	97.71 ± 1.51	99.70 ± 1.22	102.38 ± 1.08	101.10 ± 1.08	99.85 ± 1.54	98.45 ± 1.01	99.72 ± 2.05
Global recovery rate^b^ (mean ± SD)	98.66 ± 0.54	99.72 ± 1.94			101.06 ± 1.50			99.34 ± 1.54		

^a^Sample size *n* = 6. ^b^*t* values: 4.216 (raw material), 0.426 (lipstick), 2.119 (emulsion), and 1.283 (foundation). *t* critical value (0.05 significance level): 4.303 (raw material), and 2.306 (lipstick, emulsion, and foundation). ^c^No Zn was added to the raw material because its nominal concentration was assumed from the purity as declared in the certificate of analysis issued by the supplier.

**Table 6:** Youden–Steiner design for selected variables of the method.

Parameter	Experiment number							
	1	2	3	4	5	6	7	8
Ethanol (mL)	**75**	65	65	**75**	**75**	65	**75**	65
Ultrasound bath temperature (°C)	**55**	**55**	45	45	**55**	45	45	**55**
First sonication step (min)	15	15	15	**30**	**30**	**30**	15	**30**
Second sonication step (min)	15	**30**	**30**	**30**	15	15	15	**30**
Final concentration (ppm)	0.3	0.3	**0.7**	**0.7**	**0.7**	0.3	**0.7**	0.3

Bold values are the standard conditions of the method.

## Data Availability

The data used to support the findings of this study are available from the corresponding author upon request.
